# Dynamic TIMI Risk Score for STEMI

**DOI:** 10.1161/JAHA.112.003269

**Published:** 2013-02-22

**Authors:** Sameer T. Amin, David A. Morrow, Eugene Braunwald, Sarah Sloan, Charles Contant, Sabina Murphy, Elliott M. Antman

**Affiliations:** 1 Department of Cardiology, University of California at Los Angeles, Los Angeles, CA (S.T.A.); 2 Department of Cardiology, TIMI Study Group, Brigham and Women's Hospital and Harvard University, Boston, MA (D.A.M., E.B., S.S., S.M., E.M.A.); 3 Tufts Clinical and Translational Research Center, Boston, MA (C.C.)

**Keywords:** coronary artery disease, mortality, myocardial infarction, prognosis

## Abstract

**Background:**

Although there are multiple methods of risk stratification for ST‐elevation myocardial infarction (STEMI), this study presents a prospectively validated method for reclassification of patients based on in‐hospital events. A dynamic risk score provides an initial risk stratification and reassessment at discharge.

**Methods and Results:**

The dynamic TIMI risk score for STEMI was derived in ExTRACT‐TIMI 25 and validated in TRITON‐TIMI 38. Baseline variables were from the original TIMI risk score for STEMI. New variables were major clinical events occurring during the index hospitalization. Each variable was tested individually in a univariate Cox proportional hazards regression. Variables with *P*<0.05 were incorporated into a full multivariable Cox model to assess the risk of death at 1 year. Each variable was assigned an integer value based on the odds ratio, and the final score was the sum of these values. The dynamic score included the development of in‐hospital MI, arrhythmia, major bleed, stroke, congestive heart failure, recurrent ischemia, and renal failure. The C‐statistic produced by the dynamic score in the derivation database was 0.76, with a net reclassification improvement (NRI) of 0.33 (*P*<0.0001) from the inclusion of dynamic events to the original TIMI risk score. In the validation database, the C‐statistic was 0.81, with a NRI of 0.35 (*P*=0.01).

**Conclusions:**

This score is a prospectively derived, validated means of estimating 1‐year mortality of STEMI at hospital discharge and can serve as a clinically useful tool. By incorporating events during the index hospitalization, it can better define risk and help to guide treatment decisions.

## Introduction

Patients suffering from ST‐elevation myocardial infarction (STEMI) have preexisting characteristics that vary across a range of severity. This unique milieu produces post‐STEMI complications at a variable yet quantifiable rate.^1^ The process of estimating the long‐term risk of morbidity and mortality after STEMI is based on 2 levels of assessment. At hospital admission, patients can be stratified by demographics, physical examination, and presenting signs, as well as initial laboratory and angiographic data.^2–6^ The second level of assessment involves the identification of long‐term risk based on the development of postevent complications. It is well established that patients who have peri‐infarction morbidity are at increased risk for downstream events.^7^

Over the last 10 years, multiple methods of risk stratification for STEMI have been developed. The TIMI risk score,^3^ TIMI risk index,^8^ GRACE risk index,^4^ Zwolle primary PCI (percutaneous coronary intervention) risk index,^5^ and CADILLAC risk score^6^ are all prospectively validated predictors of both short‐ and long‐term mortality.^9^ Although each relies on a wide range of admission and peri‐PCI characteristics, these methods do not reclassify patients based on in‐hospital events.^3–6,8^ Patients are tied to the score they were assigned on admission without regard to their initial recovery from the index event. Although some validated risk scores include in‐hospital events, there is a need for a risk stratification method that can be easily calculable at bedside, added as an arithmetic sum, and used as an additional component of an existing scoring system.^10–12^

The wide spectrum of STEMI patients includes both those who are initially deemed high risk but have an uneventful hospital recovery as well as low‐risk patients who suffer major morbidity prior to discharge. Methods of prognostication that merge these populations do not take advantage of important information gathered in the first few days following the event.

Our primary goal is to present a new method of STEMI risk stratification that incorporates both initial assessment of risk as well as a discharge estimation of 1‐year mortality. This clinical risk score, derived from a multivariable analysis, can be calculated at the bedside and is a dynamic updating of the TIMI risk score for STEMI at the time of discharge, conditional on having survived the index hospitalization.

## Methods

We used the database of STEMI patients in Enoxaparin and Thrombolysis Reperfusion for Acute Myocardial Infarction Treatment (ExTRACT)‐TIMI 25, a study comparing enoxaparin with unfractionated heparin as an adjunctive therapy to fibrinolysis to derive the dynamic risk score for STEMI.^13^ The score was then validated using a database of STEMI patients in Trial to Assess Improvement in Therapeutic Outcomes by Optimizing Platelet Inhibition with Prasugrel (TRITON)‐TIMI 38, a clinical trial comparing prasugrel with clopidogrel in moderate‐ to high‐risk acute coronary syndrome patients scheduled for PCI.^14^ The impact of adding information about in‐hospital events was evaluated by reclassifying patients into new risk categories and assessing the performance of the new risk model.

### Derivation Set

ExTRACT‐TIMI 25 was a double‐blinded, double‐dummy trial conducted at 674 sites in more than 48 countries, randomly assigning 20 506 STEMI patients to either enoxaparin or unfractionated heparin during the index hospitalization. All patients had ≥20 minutes of ischemic symptoms while at rest and within 6 hours prior to randomization, ST‐segment elevation of ≥0.1 mV in 2 limb leads or of 0.2 mV in ≥2 contiguous precordial leads or left bundle‐branch block and were scheduled for fibrinolysis. The exclusion criteria for ExTRACT‐TIMI 25 included cardiogenic shock, pericarditis, symptoms of aortic dissection, contraindication to fibrinolysis, receipt of a low‐molecular‐weight heparin within an 8‐hour period prior to study entry, life expectancy of <12 months, and known renal insufficiency (Cr >2.5 mg/dL for men and >2.0 mg/dL for women). Patients received streptokinase, tenecteplase, altepase, or reteplase as well as 150 to 325 mg of nonenteric aspirin or 500 mg of IV aspirin unless previously received within the last 24 hours. The primary end point for the study was death or nonfatal recurrent myocardial infarction.

### Validation of the Dynamic TIMI Risk Score for STEMI

The dynamic risk score was validated in the 3534‐patient STEMI subgroup of the TRITON‐TIMI 38 trial, utilizing the 1829 patients with complete information from their index hospitalization.^14^ This trial compared prasugrel to clopidogrel by randomly assigning 13 608 patients with moderate‐ to high‐risk acute coronary syndromes with scheduled PCI to receive 1 or the other thienopyridine. The primary outcome efficacy end point was cardiovascular death, nonfatal myocardial infarction, or nonfatal stroke. STEMI patients were enrolled within 12 hours after the onset of symptoms if primary PCI was planned or within 14 days after receiving medical treatment for STEMI. Exclusion criteria included an increased risk of bleeding, anemia, thrombocytopenia, a history of intracranial pathology, or the use of a thienopyridine within 5 days of enrollment. A loading dose of study medication (either 60 mg of prasugrel or 300 mg of clopidogrel) was administered in a double‐blind fashion within an hour of PCI and subsequently continued as a maintenance daily dose (10 mg of prasugrel or 75 mg of clopidogrel). Daily aspirin use was an additional requirement.

Renal failure, congestive heart failure, and recurrent ischemia were assessed based on an investigator's acknowledgment of a serious adverse event. The 10 patients with missing information regarding the time elapsed prior to receiving a lytic in ExTRACT‐TIMI 25 were counted as having received a lytic within 4 hours.

### Statistical Analysis

The derivation of the dynamic score is based on evaluation of 1‐year mortality in ExTRACT‐TIMI 25. The model was developed using the 19 121 patients with complete information from their index hospitalization and was then reevaluated in the total population. Baseline variables were taken from the original TIMI risk score for STEMI: age, diabetes mellitus/hypertension/angina, blood pressure, heart rate, Killip class, weight, anterior ST‐elevation or left bundle branch block, and time to treatment.^3^ New in‐hospital variables assessed for inclusion in the full multivariable model came from a search of risk factors previously established as having predictive capacity after STEMI.^2,7,15–26^ These risk factors were further limited to clinical events occurring during the index hospitalization, up to day 8, which would lead to changes in outcomes or course. We have excluded patients who died during the hospitalization.

Ten new dichotomous variables were selected for testing (Table 1): recurrent myocardial infarction (MI), stroke, new Killip class III or IV congestive heart failure (CHF) or cardiogenic shock, TIMI major bleeding (including coronary artery bypass grafting [CABG] bleeding), arrhythmia (ventricular fibrillation, ventricular tachycardia, or atrial fibrillation), renal failure, performance of CABG within 8 days, recurrent myocardial ischemia, urgent revascularization, and performance of PCI within 8 days. Renal failure was an incident defined as a “serious renal failure event, either life‐threatening or resulting in disability, prolonged hospitalization, or need for renal replacement therapy” by investigators in the course of the ExTRACT‐TIMI 25 and TRITON‐TIMI 38 trials. Patients with creatinine clearance ≤30 mL/minute at baseline were counted as not developing renal failure during the index hospitalization. Recurrent myocardial infarction and recurrent myocardial ischemia are defined, CEC (clinical end point committee)–adjudicated end points in ExTRACT‐TIMI 25. Arrhythmia was defined as atrial fibrillation, ventricular tachycardia, or ventricular fibrillation.

**Table 1.** Characteristics Analyzed for Development of Dynamic TIMI Risk Score for STEMI

Characteristics	Univariate Analysis			Multivariable Analysis		
	β Coefficient	*P* Value	OR (95% CI)	β Coefficient	*P* Value	OR (95% CI)
Baseline TIMI risk score for STEMI						
Age, y						
65 to 74	0.98	<0.001	2.57 (2.31 to 3.10)			
>75	1.61	<0.001	5.04 (4.32 to 5.90)			
Diabetes mellitus	0.70	<0.001	2.01 (1.74 to 2.33)			
Hypertension	0.71	<0.001	2.04 (1.80 to 2.32)			
Angina	0.80	<0.001	2.22 (1.97 to 2.53)			
SBP <100	0.57	<0.001	1.76 (1.34 to 2.31)			
Heart rate >100	1.05	<0.001	2.84 (2.39 to 3.39)			
Killip class II to IV	1.06	<0.001	2.90 (2.50 to 3.35)			
Weight <67 kg	0.40	<0.001	1.49 (1.29 to 1.71)			
Anterior STE or LBBB	0.42	<0.001	1.51 (1.34 to 1.72)			
Time to rx >4 hours	0.07	<0.001	1.08 (1.06 to 1.10)			
Added index hospital events for dynamic score						
Recurrent MI	0.47	0.003	1.60 (1.18 to 2.19)	0.34	0.04	1.40 (1.01 to 1.94)
Stroke	2.29	<0.001	9.83 (7.33 to 13.20)	1.59	<0.001	4.90 (3.30 to 7.26)
CHF/shock	1.94	<0.001	6.95 (5.67 to 8.52)	1.16	<0.001	3.19 (2.53 to 4.01)
Major bleed	1.48	<0.001	4.39 (3.22 to 5.98)	0.34	0.09	1.41 (0.94 to 2.10)
Arrhythmia	0.81	<0.001	2.25 (1.88 to 2.69)	0.47	<0.001	1.60 (1.32 to 1.94)
Renal failure	2.28	<0.001	9.73 (4.85 to 19.52)	0.95	0.01	2.59 (1.22 to 5.50)
CABG	−0.23	0.640	0.80 (0.30 to 2.09)			
Recurrent ischemia	0.40	0.005	1.49 (1.13 to 1.97)			
Urgent revasc	−0.04	0.853	0.96 (0.60 to 1.53)			
PCI	−0.09	0.604	0.91 (0.65 to 1.28)			

TIMI indicates thrombolysis in myocardial infarction; STEMI, ST‐elevation myocardial infarction; OR, odds ratio; CI, confidence interval; SBP, systolic blood pressure; STE, ST‐elevation; LBBB, left bundle branch block; rx, treatment; MI, myocardial infarction; CHF, congestive heart failure; CABG, coronary artery bypass graft; revasc, revascularization; PCI, percutaneous interventions.

All elements of the original TIMI risk score for STEMI were retested as part of this initial assessment to reconfirm significance (Table 1). Each of the 10 additional index hospital event variables was first tested individually in a univariate Cox proportional hazards regression to determine its significance as a predictor. New variables with *P*<0.05 were incorporated into a full multivariable Cox model. Maximum likelihood estimates of the parameter coefficients were obtained using R version 2.9.1 (2009‐06‐26, The R Foundation for Statistical Computing). Each new variable was assigned a value based on the point estimate of the odds ratio (rounded to the nearest integer). The risk score categories were collapsed when the prevalence of a given score was <1%. Therefore, risk scores of 0 and 1 were combined for the purpose of the multivariable analysis. The combined risk score of 0 or 1 was used as the reference group.

The discriminatory capacity of the full multivariable model was determined by the area under the receiver operating characteristic curve (ROC) for dichotomous outcomes, adapted for survival analysis. The C‐statistic is a rank‐order statistic that reflects the concordance between prediction and outcomes. Analyses were performed using STATA/SE 10.1 (STATA Corp., College Station, TX) and R version 2.9.1 (2009‐06‐26, The R Foundation for Statistical Computing).

### Net Reclassification Improvement and Integrated Discrimination Improvement

Net reclassification improvement (NRI) was calculated to assess the change in discrimination after adding dynamic events to the TIMI risk score for STEMI. The NRI uses reclassification tables to examine whether there is an additive benefit gained from reclassifying patients into different categories based on the addition of new markers.^27^ By calculating the NRI, we were able to quantify the degree to which the new variables were driving correct movement between categories. The sum NRI was calculated by adding the net reclassification (proportion of individuals moving up minus the proportion moving down) of the event and nonevent group.^27^ An NRI can be interpreted as the percentage by which the net classification has improved with the addition of the new variables.^27^ The NRI was used to evaluate the improvement in classification obtained by adding dynamic events to the TIMI risk score for STEMI in both the ExTRACT‐TIMI 25 and TRITON‐TIMI 38 databases.

Integrated discrimination improvement (IDI) was calculated to assess the benefit of reclassification in both databases. The IDI measured enhancement in sensitivity, gained without foregoing specificity, from the addition of new variables to the TIMI risk score for STEMI.^27^ As an integral, the IDI grades the dynamic TIMI risk score's performance in improving overall sensitivity and specificity. The value obtained as the IDI can best be interpreted as the absolute increase in sensitivity given a constant specificity.

## Results

### Dynamic TIMI Risk Score for STEMI

The Cox model for death using the baseline TIMI risk score had a C‐statistic of 0.73. After this verification of the discrimination of the baseline score, 10 variables (reflecting events during the index hospitalization) were considered for inclusion in the dynamic TIMI risk score for STEMI (Table 2). Of the 10 new variables, 5 were significant when analyzed together with the baseline variables in a full multivariable model: recurrent MI, stroke, CHF/shock, arrhythmia, and renal failure. Major bleed (odds ratio, 1.41; 95% confidence interval [CI], 0.94 to 2.10) was retained in the model as the sixth variable despite a 95% confidence that encompassed the line of unity, as this was judged to be trending toward significance, hampered by insufficient power. The similarity of parameter estimates allowed us to create the new score through an arithmetic sum of the odds ratios. The dynamic score can be calculated on discharge as a simple addition to the original composite score from admission (Table 2). The treatment effect in ExTRACT‐TIMI 25 (enoxaparin superior to unfractionated heparin) remained significant, as seen in the original analyses of the data.^13^ No statistically significant (*P*<0.10) interactions were seen between treatment and score. Thus, the dynamic risk score was not acting as a surrogate for the treatment effect in the trial.

**Table 2.** Dynamic TIMI Risk Score for STEMI Summarized

	Points
Baseline TIMI risk score for STEMI	0 to 14 possible points
Age, y	
65 to 74	2
>75	3
DM/HTN/angina	1
Systolic blood pressure <100 mm Hg	3
Heart rate >100	2
Killip class II to IV	2
Weight <67 kg	1
Anterior STE or LBBB	1
Time to rx >4 hours	1
Added index hospital events for dynamic score	
Recurrent MI	1
Stroke	5
Major bleed	1
CHF/shock	3
Arrhythmia	2
Renal failure	3
Dynamic TIMI risk score	0 to 29 possible points*

TIMI indicates thrombolysis in myocardial infarction; STEMI, ST‐elevation myocardial infarction; DM, diabetes mellitus; HTN, hypertension; STE, ST‐elevation; LBBB, left bundle branch block; rx, treatment; recurrent MI, recurrent myocardial infarction; CHF, congestive heart failure.

* Baseline TIMI risk score for STEMI has 0 to 14 possible points.

Whereas the baseline TIMI risk score for STEMI has 0 to 14 possible points, the dynamic TIMI risk score has a total of 0 to 29 possible points, with 0 to 15 points assigned based on in‐hospital events (Table 2).

**Figure 1.**
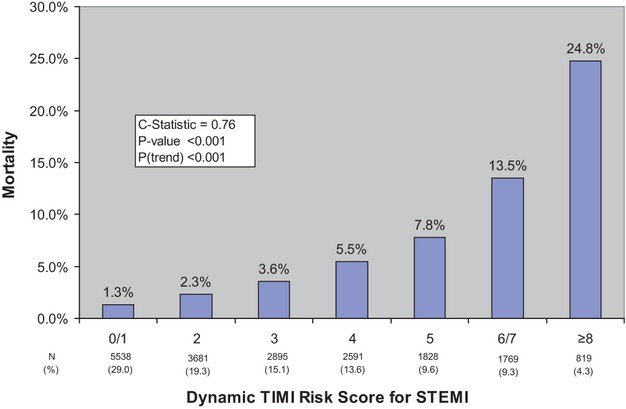
Dynamic TIMI risk score for STEMI used to predict 1‐year mortality. Data are based on the ExTRACT‐TIMI 25 derivation set. N refers to the number of patients falling under that score category, whereas % refers to the percentage of the total population in that score category. TIMI indicates Thrombolysis In Myocardial Infarction; STEMI, ST‐elevation myocardial infarction.

The dynamic TIMI risk score has a high discriminatory capacity, with a C‐statistic of 0.76, measured by the area under the ROC curve. There is a strong association with 1‐year mortality. The distribution of the score, which has a range from 0 to 29, was examined. Scores 0 to 2 and all scores ≥8 were collapsed into a single category because of the small number of study subjects in the individual score categories. As the dynamic risk score increases, the 1‐year mortality rose (Figure 1). There is an approximate 25% absolute increase in 1‐year mortality after discharge between a score of 0/1 and ≥8, with a *P* trend of *P*<0.001 by χ^2^ for trend.

### Validation of Dynamic TIMI Risk Score

**Figure 2.**
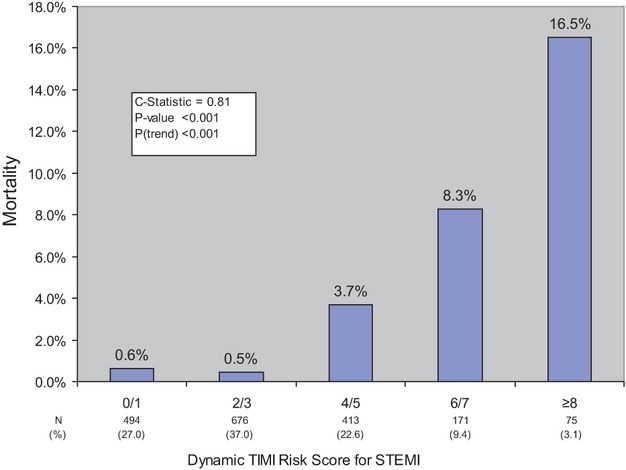
Dynamic TIMI risk score for STEMI for predicting 1‐year mortality in the TRITON‐TIMI 38 validation set. N refers to the number of patients falling under that score category, whereas % refers to the percentage of the total population in that score category. Risk categories were collapsed because of the low prevalence of patients assigned to certain scores. TIMI indicates Thrombolysis In Myocardial Infarction; STEMI, ST‐elevation myocardial infarction.

The dynamic TIMI risk score was validated using a database of 3534 STEMI patients in TRITON‐TIMI 38 as an external data set. Unlike the patients in ExTRACT‐TIMI 25, 99% of this population underwent PCI. The treatment effect in TRITON‐TIMI 38 remained significant, as seen in the original analyses of the data.^14^ No statistically significant (*P*<0.10) interactions were seen between treatment and score. The predictive capacity of the dynamic TIMI risk score remained consistent for 1‐year mortality, with a C‐statistic of 0.81. As with the derivation data, the validation set demonstrated increasing mortality with escalating risk score (Figure 2).

### Net Reclassification Improvement and Integrated Discrimination Improvement

The reclassification benefit of the dynamic TRS was assessed by monitoring movement between low‐, moderate‐, and high‐risk (defined as <5% 1‐year mortality for low risk, 5% to 15% for moderate risk, and >15% for high risk) categories (Table 3). In the ExTRACT‐TIMI 25 database, 5411 patients were assigned a moderate risk of 1‐year mortality by the baseline TRS for STEMI.Using the dynamic risk score, 3911 of these patients were reclassified under the dynamic TRS model, 176 into the high‐risk category, and 3815 into the low‐risk category (Table 3). Among the 925 patients assigned to a high‐risk category by the baseline TIMI risk score, 639 were lowered to moderate risk based on the dynamic TRS. Very few of the patients assigned a low risk by the TRS were reclassified as moderate‐ (156) or high risk (34). In total, 25.2% of the patients in ExTRACT‐TIMI 25 experienced a change in risk category because of the addition of in‐hospital events. The largest change in score was 6 points, whereas the median change in score for those who were reclassified was 2. The maximum change in estimated 1‐year mortality for a patient was 30%.

**Table 3.** Reclassification Among Risk Groups for TRS Versus Dynamic TRS in ExTRACT‐TIMI 25

TRS for STEMI	Total	Dynamic TRS for STEMI		
		Low Risk*	Moderate Risk*	High Risk*
Low risk*	12 785	12 595	156	34
Moderate risk*	5411	3815	1420	176
High risk*	925	4	639	282

TRS indicates TIMI risk score; TIMI, thrombolysis in myocardial infarction; STEMI, ST‐elevation myocardial infarction. Data shown are indicative of the number of patients.

* <5% Mortality.

* 5% to 15% Mortality.

* >15% Mortality.

The net reclassification of subjects improved using the model for assessing dynamic risk. In ExTRACT‐TIMI 25, the dynamic risk score produced a net reclassification improvement of 0.33 with *P*<0.0001 over the original risk score, that is, a 33% improved classification. In the TRITON‐TIMI 38 database, the NRI was 0.35 with *P*=0.01. Therefore, in the validation set, the dynamic score provided a net reclassification improvement of approximately 35% over the original risk score for STEMI.

The IDIs calculated from ExTRACT‐TIMI 25 and TRITON‐TIMI 38 demonstrated that the dynamic TRS provides a statistically significant improvement in sensitivity and specificity. The IDI for the new risk score in ExTRACT‐TIMI 25 was 0.019 (*P*<0.0001). The IDI of 0.050, calculated from TRITON‐TIMI 38, represented a statistically significant benefit (*P*=0.049) because of the addition of dynamic factors to the original TIMI risk score for STEMI.

### Illustration of Use of Dynamic Risk Score

By the addition of variables from the entire course of the index hospitalization (up to 8 days), the dynamic risk score provides a better ability to predict outcome, estimating a more accurate 1‐year mortality for each individual. This concept is demonstrated in Table 4. In this illustration, patient 1 is admitted to the hospital with a STEMI and is assessed a baseline TIMI risk score of 4, estimating a 1‐year mortality of 7.10% on admission. The dynamic TRS, assessed on hospital discharge, assigns additional points for in‐hospital events. If the patient were to experience congestive heart failure and atrial fibrillation during the index hospitalization, 5 additional points would be assigned, resulting in an updated 1‐year mortality of 24.80% (3.49‐fold increase in risk). Such dynamic updating reassigns the patient from a moderate‐risk to a high‐risk category. A patient who does not experience any in‐hospital events has a lower updated 1‐year mortality compared with that predicted by the baseline TIMI risk score on admission. Patient 2 is admitted to the hospital with a similar baseline TRS of 4 and an estimated 1‐year mortality of 7.10%. The patient does not experience an in‐hospital event and is discharged with a dynamic TRS of 4 and an updated 1‐year mortality of 5.50% (representing 0.77 the risk as predicted from baseline TIMI risk score). An uneventful hospital course lowers predicted 1‐year mortality for every score. Alternatively, a patient may experience an in‐hospital event with little change in estimated mortality. For example, patient 3 had a similar baseline TRS of 4 with an initial estimated 1‐year mortality of 7.10%. If the patient experiences a major bleed during the course of the hospitalization, the updated 1‐year mortality is 7.80% (1.09‐fold increase in risk compared with baseline).

**Table 4.** Clinical Examples of the Dynamic TRS

Example Patient	Baseline TRS	Estimated 1‐Year Mortality*, %	In‐Hospital Events	Added Points*	Dynamic TRS	Updated 1‐Year Mortality*, %	Fold Change in Risk Compared With Baseline
1	4	7.10	CHF, arrhythmia	5	9	24.80	3.49
2	4	7.10	None	0	4	5.50	0.77
3	4	7.10	Major bleed	1	5	7.80	1.09

TRS indicates TIMI (thrombolysis in myocardial infarction) risk score; CHF, congestive heart failure.

* Based on TRS for STEMI.

* As dictated by dynamic TRS for STEMI.

* Based on dynamic TRS for STEMI, conditional on having survived to hospital discharge.

## Discussion

The dynamic TIMI risk score for STEMI is a prospectively validated means of prognostication that incorporates an accepted tool, the TIMI risk score, to allow a continuous assessment of risk. Risk stratification is a dynamic process, altered by a patient's changing condition and informed by each preceding complication.^7,23^ Therefore, a more complete scoring system should deliver an initial estimate of disease severity and then incorporate in‐hospital events prior to rendering a long‐term mortality estimate.

Our results have generated a classification system that can be easily calculated at the bedside, has been validated in a large unrelated cohort, and takes into account the dynamic nature of risk assessment. The TIMI risk score can be used in its original form to evaluate historical information, physical examination, and details of the presentation to provide clinicians with a measurement of initial disease severity for patients entering the hospital, through prediction of their potential in‐hospital and 30‐day mortality.^3^ The 6 elements of the dynamic score occurring during the index hospitalization can then be added to the admission score to produce an estimate of 1‐year mortality on discharge (Table 2). This information may then be used by the discharging physician, outpatient clinician, and patient to help steer subsequent care.

A number of helpful stratification schemes are currently in use, many of them focused on supplying prognostic information at presentation.^3–6,8^ Although some validated risk scores include in‐hospital events, none allow a physician to assess risk at admission and then easily reclassify on discharge while retaining the simplicity of an adjunctive scoring system.^4,10–12^ The dynamic TIMI risk score for STEMI retains all the benefits of the original TRS including the absence of weighted terms, the simplicity of integer values, and the ability to produce a final score through simple addition. Furthermore, the dynamic score is fully compatible with the original, allowing a risk assessment on both admission and discharge. The dynamic risk stratification described here provides the benefit of a highly accurate estimation of 1‐year mortality and an excellent discriminatory capacity without the need for computer calculation. It can easily be calculated by either physicians or physician extenders. With the needs of underresourced medical settings in mind, the dynamic risk score has been developed without novel markers or laboratory values. All the score's elements are easily recognizable clinical events that do not require prognostic tests.

A dynamic risk score that can provide both an initial risk assessment and subsequent discharge reclassification could help clinicians to make decisions about the postdischarge care of STEMI patients. Physicians caring for patients post‐STEMI could use the dynamic TRS to inform frequency of follow‐up and decide on the threshold for a monitored trial of treatment withdrawal. The risk‐benefit ratio for the use of therapeutic devices and drugs varies with a patient's estimated mortality and morbidity.^28–29^ Without a detailed understanding of the patient's risk at discharge, it is difficult to evaluate the efficacy of new treatments. Also, treatments such as prasugrel or ticagrelor may have enhanced absolute and relative benefits when there is a higher estimated mortality.^29^ The dynamic TRS can better define risk, perhaps guiding tailored cardiopulmonary rehabilitation, frequency of follow‐up visits, and consideration of potent but expensive therapeutics for targeted populations.^30–33^

In addition, the dynamic TRS could be used by investigators planning future randomized controlled trials to target appropriate populations for hypothesis testing. It could help to define a higher‐risk cohort for whom more intense therapy can reduce mortality (eg, adding a new oral anticoagulant on top of current therapy). Alternatively, the dynamic TRS may be useful in targeting lower‐risk patients who would be good candidates for withdrawal of therapies after a finite post‐MI period. Because innovation in post‐STEMI care has been additive, most commonly used medications have not been tested in the setting of modern treatments. By identifying low‐risk patients, we can begin a more contemporary assessment of post‐STEMI treatments. Such analyses would be particularly relevant in our current environment of restrictive economics.

As with any attempt to balance the need for simplicity versus the desire for accuracy, this study has its limitations. The proportionality assumption was examined for the new in‐hospital variables. The only new in‐hospital variable that violated the proportional hazard assumption was renal failure. We believe that in this case the proportional hazard test is probably unstable because of the small number of subjects with renal failure (n=17). It is widely accepted that the Cox proportional hazards model is robust to small proportionality assumption violations.^34^ The overall hazard ratio for renal failure can be interpreted as an average effect over time.

In addition, it should be noted that the derivation and validation of the dynamic risk score has been conducted in phase 3 studies. The score was derived in a majority fibrinolysis‐treated population from ExTRACT‐TIMI 25 and found to be translatable to an entirely PCI‐treated population in TRITON‐TIMI 38. Because the score was developed using patients who qualified for enrollment, application to a variety of medical settings must still be assessed.^35–36^ Although absolute risk prediction based on the derivation data set may not be applicable to all populations, the score's ability to risk‐stratify has been reliably demonstrated in the TRITON‐TIMI 38 external population. The score has been validated only for use with STEMI patients and may not be able to reliably predict risk at alternate times or for populations with unstable angina and NSTEMI. The decision to exclude novel markers and laboratory studies while focusing on in‐hospital clinical events was consciously made to allow for wide employment of the score without the need for further testing. Although the exclusion was deliberate, factors such as troponin elevation and lack of ST‐elevation resolution have been shown to confer risk and may have a significant independent effect on mortality.^37–40^

It is also important to note that we have used the original TRS as a comparator when assessing the performance of the new score. Although the original TIMI risk score for STEMI was derived for short‐term mortality, the dynamic TRS has only been derived and validated for 1‐year outcomes. The dynamic TRS updates the risk for patients who survive to discharge and allows for reclassification based on in‐hospital events. In essence, the new model is predictive of a new outcome not assessed by the original TRS. The decision was made to use the original TRS as the basis for comparison because it shares a common derivation and continues to have a strong discriminatory capacity for prediction of 1‐year mortality.^8^

## Conclusions

Risk stratification of STEMI patients is a continuous process.^23^ Over time, a patient's condition can change, altering estimated mortality. The dynamic TIMI risk score for STEMI is a prospectively derived, validated means of updating the estimate of 1‐year mortality at the time of hospital discharge. Unlike baseline risk assessments, this score incorporates information from events during the index hospital course that are known to affect mortality risk from STEMI after hospital discharge.

## Acknowledgments

Dr Elliott Antman had full access to all the data in the study and takes responsibility for the integrity of the data and the accuracy of the data analysis. *Study concept and design:* Amin, Morrow, Braunwald, Antman. *Acquisition of data:* Amin, Antman, Sloan, Murphy, Contant. *Analysis and interpretation of data:* Amin, Antman, Sloan, Murphy, Contant. *Drafting of the manuscript:* Amin, Morrow, Braunwald, Antman. *Critical revision of the manuscript for important intellectual content:* Amin, Morrow, Braunwald, Antman. *Statistical analysis:* Amin, Antman, Sloan, Murphy, Contant. *Administrative, technical, or material support:* Morrow, Braunwald, Antman. *Study supervision:* Morrow, Braunwald, Antman.
